# Carlevale Versus Yamane Sutureless Scleral Fixation: Clinical and Swept-Source AS-OCT Comparative Analysis in Eyes Without Capsular Support

**DOI:** 10.3390/jcm15218511

**Published:** 2026-11-02

**Authors:** Erik Mus, Antonio Valastro, Maura Mancini, Edoardo Bordon, Emanuele Koumantakis, Fabio Battaglino, Christine Rollandin, Luca Ventre

**Affiliations:** 1 Department of Ophthalmology, Beauregard Hospital, Azienda USL della Valle d’aosta, Via L. Vaccari 5, 11100 Aosta, Italy; erikuniversita@gmail.com (E.M.);; 2 Department of Biomedical Sciences, Ophthalmology Clinic, University of Messina, 98121 Messina, Italy; 3 Faculty of Medicine, University of Turin, 10126 Turin, Italy; 4 Center for Biostatistics, Epidemiology, and Public Health, Department of Clinical and Biological Sciences, University of Turin, 10043 Orbassano, Italy; 5 Cancer Epidemiology Unit, University Hospital Città della Salute e della Scienza, 10126 Turin, Italy; 6 Structure Preventive Medicine and Risk Management, Strategic Management of the Local Health Authority of Valle d’Aosta, 11100 Aosta, Italy

**Keywords:** anterior segment optical coherence tomography (AS-OCT), Intra Ocular Lens (IOL), intraoperative complications, postoperative complications, sutureless scleral fixated IOL (SF-IOL)

## Abstract

**Purpose:** We aimed to compare the anatomical and functional outcomes, postoperative complications, and intraocular lens (IOL) positioning of two different techniques of sutureless scleral fixation: the Yamane double-needle flanged technique and the Carlevale intraocular lens. **Methods:** This retrospective, single-center, comparative observational study included 100 eyes of 100 patients who underwent secondary scleral-fixated IOL implantation between January 2021 and December 2024. Sixty-nine eyes underwent Yamane fixation and 31 eyes received a Carlevale IOL. Best-corrected visual acuity (BCVA), postoperative complications, and anatomical stability were evaluated. In a subgroup of 33 patients, IOL tilt and decentration were objectively assessed at 6 months using second-generation swept-source anterior segment optical coherence tomography (AS-OCT; Casia 2, Tomey, Nagoya, Japan). **Results:** Both techniques provided significant visual improvements compared with baseline. AS-OCT demonstrated comparable anatomical stability between groups, with mean tilt values of 6.9° and 6.4° and mean decentration values of 0.61 mm and 0.48 mm for the Yamane and Carlevale groups, respectively, without statistically significant differences. Cystoid macular edema was observed numerically more often in the Yamane group (23.1% vs. 15.4% at 6 months; *p* = 0.604), and anterior chamber blood Tyndall effect was recorded respectively in 13% vs. 6% of eyes; these differences were not statistically significant. Early postoperative complications were generally mild and self-limiting in both groups. **Conclusions:** Both the Yamane technique and the Carlevale IOL were associated with significant visual improvement and acceptable safety profiles for secondary sutureless scleral fixation in eyes without adequate capsular support. In this sample the Carlevale IOL showed a trend toward better centration, whereas second-generation swept-source AS-OCT proved to be a valuable objective imaging modality for the postoperative evaluation of scleral-fixated IOLs. Larger prospective multicenter studies are needed to confirm these findings.

## 1. Introduction

Secondary intraocular lens (IOL) implantation remains a challenging surgical procedure in eyes lacking adequate capsular support, a condition commonly encountered after complicated cataract surgery, spontaneous late in-the-bag IOL dislocation, trauma, or in systemic disorders such as Marfan syndrome and pseudoexfoliation syndrome [1].

In such cases, placement of an IOL in the capsular bag or ciliary sulcus is not feasible, and alternative fixation strategies must be considered. Several surgical options have been developed, including anterior chamber IOLs (ACIOLs), iris-claw IOLs and scleral-fixated IOLs (SF-IOLs) [2].

Among these, scleral fixation has gained increasing popularity, particularly with the advent of sutureless intrascleral fixation techniques. Traditional sutured fixation, using 9-0 or 10-0 polypropylene, has been associated with late suture degradation, IOL dislocation, and suture-related complications. In contrast, sutureless approaches provide stable long-term fixation while avoiding suture-related drawbacks [3,4,5].

The double-needle flanged intrascleral fixation technique, described by Yamane et al. in 2017, has become widely adopted due to its relative technical simplicity and reproducible results [6]. It employs a three-piece posterior chamber IOL, with haptics externalized and cauterized to create flanges that secure the lens within scleral tunnels. Despite its advantages, tilt and decentration remain potential concerns due to the lack of intrinsic haptic stabilization [6].

The Carlevale IOL (FIL-SSF, Soleko IOL Division, Pontecorvo, Italy, or Carlevale IOL High tech, MD Tech, Casoria, Italy) represents a novel single-piece acrylic lens specifically designed for sutureless scleral fixation. Its T-shaped haptic plugs are externalized beneath scleral flaps, providing secure fixation without sutures or cauterization. Early clinical studies have demonstrated favorable visual outcomes, reduced surgical variability, and promising long-term stability [7,8,9].

Anterior segment optical coherence tomography (AS-OCT) enables precise, non-invasive evaluation of IOL position, including tilt and decentration, offering valuable insights into the anatomical stability of scleral-fixated IOLs [10]. However, comparative data between the Yamane technique and the Carlevale IOL remain limited.

Besides anatomical stability, postoperative visual recovery and complication rates have become increasingly relevant outcome measures when evaluating secondary scleral-fixated IOL implantation techniques. Cystoid macular edema, intraocular pressure fluctuations, corneal edema, and long-term retinal complications may significantly affect functional recovery and overall patient satisfaction. Although several studies have separately investigated the Yamane technique and the Carlevale IOL, direct comparisons simultaneously evaluating visual outcomes, postoperative complications, and objective anatomical positioning remain scarce. Accurate and stable IOL positioning is also relevant for refractive predictability. Even after uncomplicated cataract surgery, only 73.7% of patients achieve a refractive prediction error within ±0.5 D [11], and this accuracy may be further challenged in eyes without capsular support, in which the effective lens position is less predictable and may be affected by IOL tilt and decentration.

The present study was therefore designed to perform a direct comparative analysis of these two widely adopted sutureless scleral fixation techniques, starting from the visual outcome and postoperative complications, and finally IOL tilt and decentration. By applying AS-OCT, we aimed to obtain high-resolution, objective, and reproducible measurements of tilt and decentration, parameters that are critical for the optical performance and long-term stability of IOLs.

This study differs from previous reports in several respects. Firstly, all procedures were performed by a single experienced vitreoretinal surgeon, which minimizes inter-surgeon variability, a recognized source of heterogeneity in studies on scleral fixation. Secondly, functional outcomes, postoperative complications (including OCT-detected cystoid macular edema) and objective IOL positioning were evaluated together within the same cohort. Thirdly, IOL tilt and decentration were quantified using a swept-source AS-OCT system whose built-in software models the IOL position over 360°, reducing operator-dependent variability.

## 2. Material and Methods

This was a retrospective, monocentre, comparative observational single-surgeon study. Consecutive eyes undergoing secondary IOL implantation (due to spontaneous late in-the-bag IOL dislocation or aphakia after complicated cataract surgery) were included from January 2021 to December 2024 in the Department of Ophthalmology of Beauregard Hospital (Azienda USL della Valle d’Aosta, Aosta). All the procedures were performed by only one vitreoretinal surgeon (L.V.). All study procedures adhered to the Tenets of the Declaration of Helsinki.

Exclusion criteria were history of blunt non-penetrating and perforating trauma, buckling surgery, corneal ectasia or degeneration, open globe trauma and amblyopia.

Relevant clinical data of the study population were drawn from electronic medical records. All subjects had lens-related issues with inadequate capsular support and needed scleral-fixated IOL implantation. All patients were informed about the risks and benefits of the surgery, and a written informed consent for the surgical procedure was obtained.

Only eyes with complete preoperative ophthalmological exams and postoperative evaluations after two weeks were included in the study. Preoperative and postoperative ophthalmic evaluation included age; sex; best-corrected visual acuity (BCVA); intraocular pressure (IOP); axial length (AL); complication events; type of scleral fixation IOL implantation: Carlevale IOL or 3-piece IOL scleral fixation using the Yamane technique; type of vitrectomy performed: anterior, 23G, 25G, 27G and anterior and posterior segment slit lamp examinations and fundus exams after mydriasis. Patients were classified as phakic, aphakic, or pseudophakic based on their preoperative refractive status. Patients for whom complete data were not available were excluded.

Preoperative calculation of the intraocular lenses (IOLs) was performed for both groups using the SRK/T formula. BCVA was measured with Snellen charts at each visit and converted to logMAR for the analysis. IOP was measured with Goldmann applanation tonometry and axial length was measured with optical biometer (IOLMaster 700, Zeiss Medical Technology, Oberkochen, Germany). Macular OCT was performed with posterior segment OCT (Spectralis, Heidelberg Engineering, Heidelberg, Germany); central retinal thickness (CRT) was used to define CME as the presence of intraretinal cystic fluid spaces on macular OCT (with central retinal thickening), including small or subclinical cystic changes, irrespective of their effect on visual acuity. Preoperative macular OCT was used to exclude pre-existing intraretinal fluid; only new-onset postoperative changes were classified as CME. The same definition was applied to all scans. OCT images were graded by F.B, A.V, E.B, E.M and L.V.; grading was retrospective and not masked to the surgical technique. Because this definition includes subclinical cases, the rates reported here refer to OCT-detected CME. Endothelial cell density (ECD) and central corneal thickness (CCT) were measured with a specular microscope (EM-4000, Tomey, Nagoya, Japan) at baseline to compare the corneal status of the two groups.

Follow-up visits were scheduled at 1 day, 1 week, 1 month, and 3, 6, 12 and 24 months after surgery, and data were retrieved from electronic medical records. The postoperative topical regimen consisted of a fixed combination of dexamethasone and netilmicin (1 drop four times daily for 14 days) plus bromfenac (1 drop twice daily for 14 days).

Postoperative complications, i.e., corneal edema, clinical IOL tilt or decentration, macular edema, vitreous hemorrhage, endophthalmitis, choroidal detachment, hypotony or hypertension, conjunctival erosion and retinal tear or detachment, were evaluated. Hypotony was considered a postoperative complication when the intraocular pressure (IOP) was below 6 mmHg. Ocular hypertension was considered a postoperative complication when IOP was higher than 25 mmHg.

**Figure 1:**
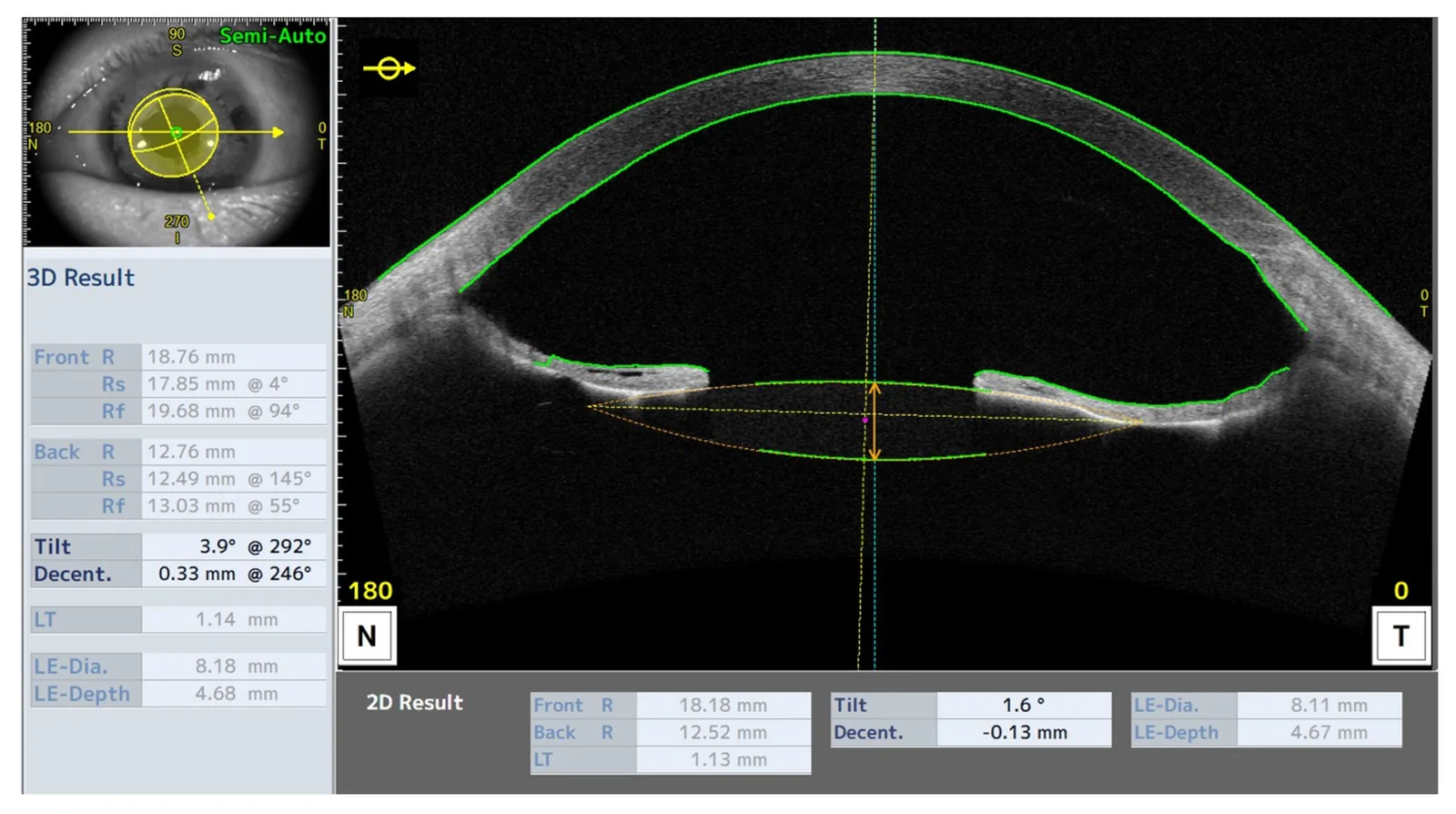
CASIA 2 display screen illustrating the integrated software analysis of intraocular lens (IOL) tilt and decentration (automated and semi-automated), shown here for scleral-fixated IOLs.

Swept-source AS-OCT was performed in a subgroup of patients 6 months after surgery, using the second-generation CASIA2 OCT (Tomey, Nagoya, Japan). Examinations were performed by an experienced operator (F.B.) with pharmacological pupil dilation, while the patient fixated on the internal target of the device. IOL decentration and tilt were measured using the inbuilt automatic software of the Casia 2 AS-OCT analyzing and modelling the IOL position over 360 degrees (see Figure 1), eliminating the need for operator-dependent post-processing of images to calculate IOL tilt and decentration. When automatic recognition of either surface was not successful, the IOL profile was segmented semi-automatically by manually placing three points on the anterior and three points on the posterior IOL surface. The calculation of tilt and decentration was then performed automatically by the software. Therefore, the manual step was limited to delineating the IOL profile, while the computation of the parameters remained fully automated, thus minimizing operator-dependent variability.

### 2.1. Surgery

All surgeries were performed under complete aseptic precautions by a highly experienced surgeon (L.V.) with patients under retrobulbar anesthesia. To reduce the influence of the learning curve, the first 30 cases performed by the surgeon (L.V.) with each technique (Yamane and Carlevale) were excluded. The IOLs used for each group were, respectively, the Carlevale IOL (FIL SSF Monofocal, SOLEKO SPA, Pontecorvo, Italy, or the Carlevale IOL hi-tech, MD Tech, Casoria, Italy) and a 3-piece IOL with haptics in PVDF (AvanseePresetUV PU6AS, Kowa, Nagoya, Japan).

**Transscleral Yamane Implantation Technique**. The three-piece IOL, when present, was unleashed from the capsular bag and prepared to be implanted using the Yamane transscleral technique. One of the haptics was extruded from the main incision to avoid a subluxation of the lens itself. This sutureless intrascleral three-piece IOL fixation is a technique also known as flanged IOL fixation and was described by Yamane in 2017 [6]. When a single-piece IOL was present, the lens was folded on itself using a nucleus manipulator with an olive shaped-tip (Janach, Como, Italy) and explanted from the main incision itself. Three-piece IOL (PU6AS, Kowa, Nagoya, Japan) was injected in the anterior chamber leaving the trailing haptic out of the main incision to avoid the lens drop in the vitreous cavity. This three-piece IOL has haptics in polyvinylidene fluoride (PVDF) and an A constant of 119.0 (SRK/T). An angled sclerotomy was made through the conjunctiva using a 30-gauge thin-wall needle at 2 mm from the limbus. The leading haptic was threaded into the lumen of the needle using forceps. A second sclerotomy then was made with a 30-gauge thin-wall needle that was 180° from the first sclerotomy. The trailing haptic was inserted into the lumen of the second needle, while the first needle was put on the eye lid. Both haptics were externalized onto the conjunctiva using the double-needle technique. The ends of the haptics were cauterized using an ophthalmic cautery device to make a flange with a diameter of 0.3 mm. The flange of the haptics was pushed back and fixed into the scleral tunnel.

**Carlevale’s Lens Implantation Technique**. Carlevale’s lens (SOLEKO FIL SSF, Soleko, Italy) is a single piece, 25% water hydrophilic acrylic IOL, with a 6.5 mm optic, a 13.2 mm diameter, and 10° vaulted haptic with a retina vault and retina convexity. Carlevale IOL has a correct direction of the implant, indicated by the presence of two small tags on the haptics and a harpoon for sutureless transscleral fixation. IOL’s power range is between −5 and +35 diopters, and the A constant is 118.7 (SRK/T).

The Carlevale IOL high-tech lens (MD Tech, Casoria, Italy) is a single-piece hybrid material IOL (hydrophilic/hydrophobic copolymer), with a 6.5 mm optic and a 13.4 mm total diameter. IOL’s power range is between +10 and +30 diopters, and the A constant U/S is 118.0.

After corneal white to white diameter evaluation (WTW), the infusion line is positioned at inferotemporal quadrant. A limited conjunctival peritomy, 2 partial 4 × 4 mm thickness scleral flaps were made and hinged at the limbus 180° apart. Then, two sclerotomies using a 25-gauge needle were placed at 2.5 mm from the limbus in correspondence to the three and nine o’clock position. The Carlevale IOL was injected into the anterior chamber through a corneal tunnel, and the leading plug was grasped with crocodile tip forceps inserted into the vitreous chamber through the sclerotomy and then externalized under the scleral flap in a single maneuver. Then, the trailing plug was grasped and externalized with 2 forceps using the handshake technique; IOL centration was achieved without performing extra-intraoperative maneuvers. Scleral flaps and conjunctival wound were sealed with nylon 10/0 and Vycril 7/0, respectively. Lastly 10/0 nylon stitch is positioned on the main incision suture.

### 2.2. Statistical Analysis

Continuous variables are presented as median and interquartile range (IQR). Qualitative variables are shown as absolute frequencies and percentages (%). Longitudinal trends in BCVA, IOP and CRT were visualized separately for each surgical group and for the overall cohort, at the following timepoints (when available): baseline, 1 day, 1 week, 1 month, 3, 6, 12, and 24 months. For each group and timepoint, the mean value and the corresponding 95% confidence interval (CI) were calculated. Longitudinal data were analyzed as available, without imputation of missing values; the number of eyes with available data decreases at later timepoints. Differences between surgical interventions were assessed using the Mann–Whitney U test for quantitative variables and Fisher’s exact test for qualitative variables.

The change in BCVA at 1 week and 1 month postoperatively (ΔlogMAR, calculated as postoperative minus baseline logMAR, with negative values indicating improvement) was analysed using multivariable linear regression. The main independent variable was the surgical technique (Yamane as the reference category). Models were adjusted for pre-specified potential confounders, i.e., age, axial length and baseline BCVA, as these may have influenced the surgeon’s choice of technique (confounding by indication). Results are reported as regression coefficients, corresponding to the unadjusted/adjusted mean difference in ΔlogMAR, with 95% CIs and two-sided *p*-values. *p*-values < 0.05 were considered statistically significant. All analyses were performed using R software version 4.6.0 [12]. Plots were generated using the “ggplot2” package (version 4.0.3).

Multivariable adjustment was restricted to the BCVA outcomes, for which the data were sufficient for a stable model. For CME and other complications (limited number of events) and for the AS-OCT analysis (limited number of eyes), multivariable models including three covariates were considered unreliable owing to the risk of overfitting and were therefore not fitted; these comparisons are unadjusted and should be interpreted as exploratory. No formal a priori sample size calculation was performed, given the retrospective design: all consecutive eligible eyes were included.

### 2.3. Use of Generative AI Tools

A generative AI tool (Claude Opus 5, Anthropic) was used to edit the English wording, translate and rephrase text drafted by the authors, and check the internal consistency of numerical values, figure callouts and reference numbering. The tool was not used to generate, analyze or interpret clinical data, to perform statistical analyses, or to produce figures. All data and scientific interpretations were provided by the authors, who reviewed and edited all AI-assisted output and take full responsibility for the content of this publication.

## 3. Results

### 3.1. Patient Demographics and Preoperative Data

The study included 100 eyes of 100 patients who underwent secondary sutureless intraocular lens (IOL) scleral fixation. The cohort was divided into two groups: Group 1 (Yamane technique) comprising 69 eyes and Group 2 (Carlevale IOL) comprising 31 eyes.

Statistical analysis of baseline characteristics revealed a significant difference in the median age of the two groups: 79.0 [74.0, 84.0] years for the Yamane group compared to 65.0 [56.0, 76.5] years for the Carlevale group (*p* < 0.001). Furthermore, the median axial length (AL) was significantly shorter in the Yamane group (23.4 [22.7, 24.6] mm) than in the Carlevale group (26.3 [23.8, 30.4]; *p* < 0.001). There were no significant differences regarding gender distribution (*p* = 0.829), mean endothelial cell density (ECD; *p* = 0.438), central corneal thickness (CCT; *p* = 0.234) or baseline intraocular pressure (IOP; *p* = 0.455).

### 3.2. Surgical Indications

**Figure 2:**
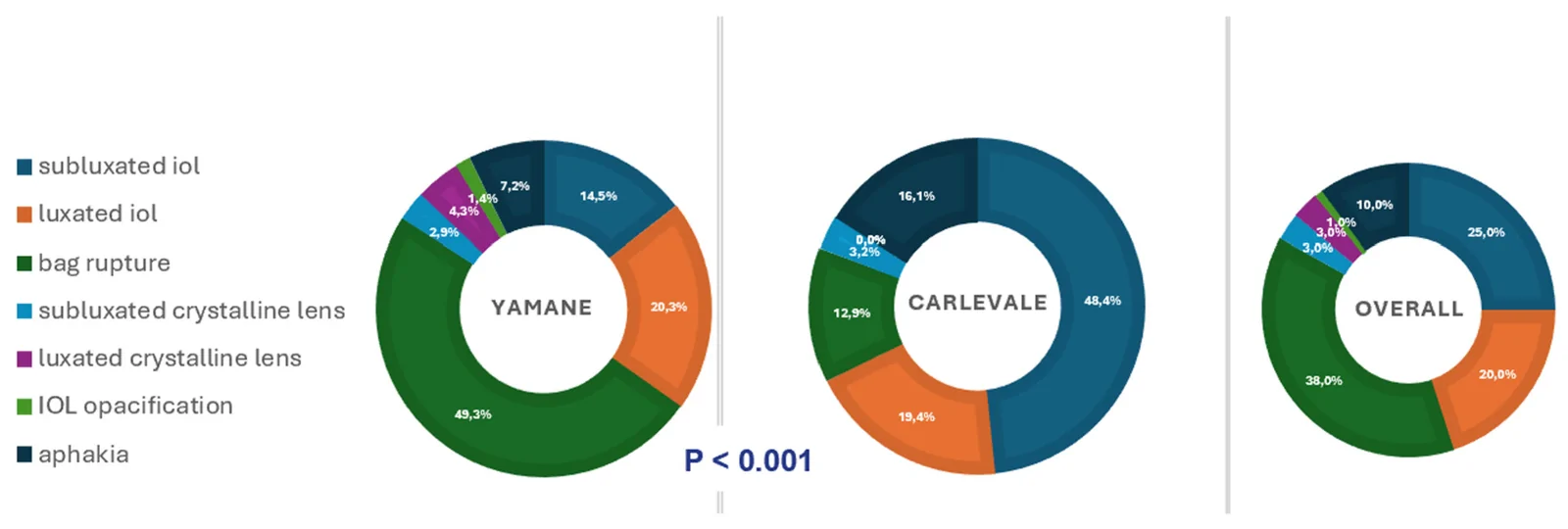
Surgical indications for secondary scleral-fixated intraocular lens (IOL) implantation. Donut charts present the percentage breakdown of clinical indications—including subluxated/luxated IOLs, posterior capsule bag rupture, subluxated/luxated crystalline lenses, IOL opacification, and aphakia—for the Yamane group, Carlevale group, and total cohort (Overall).

The clinical indications for secondary IOL fixation differed significantly between the two cohorts (*p* < 0.001). In the Yamane group, the primary indication was capsular bag rupture during cataract surgery (49.3%), followed by spontaneous IOL luxation (20.3%). Conversely, the Carlevale group was most frequently indicated for IOL subluxation (48.4%), followed by IOL luxation (19.4%) and surgical aphakia (16.1%; Figure 2). Notably, the necessity for IOL implantation following a complicated cataract surgery was reported in 52.2% of Yamane cases compared to 12.9% of Carlevale cases (*p* < 0.001).

### 3.3. Anatomical Stability: AS-OCT Evaluation

**Figure 3:**
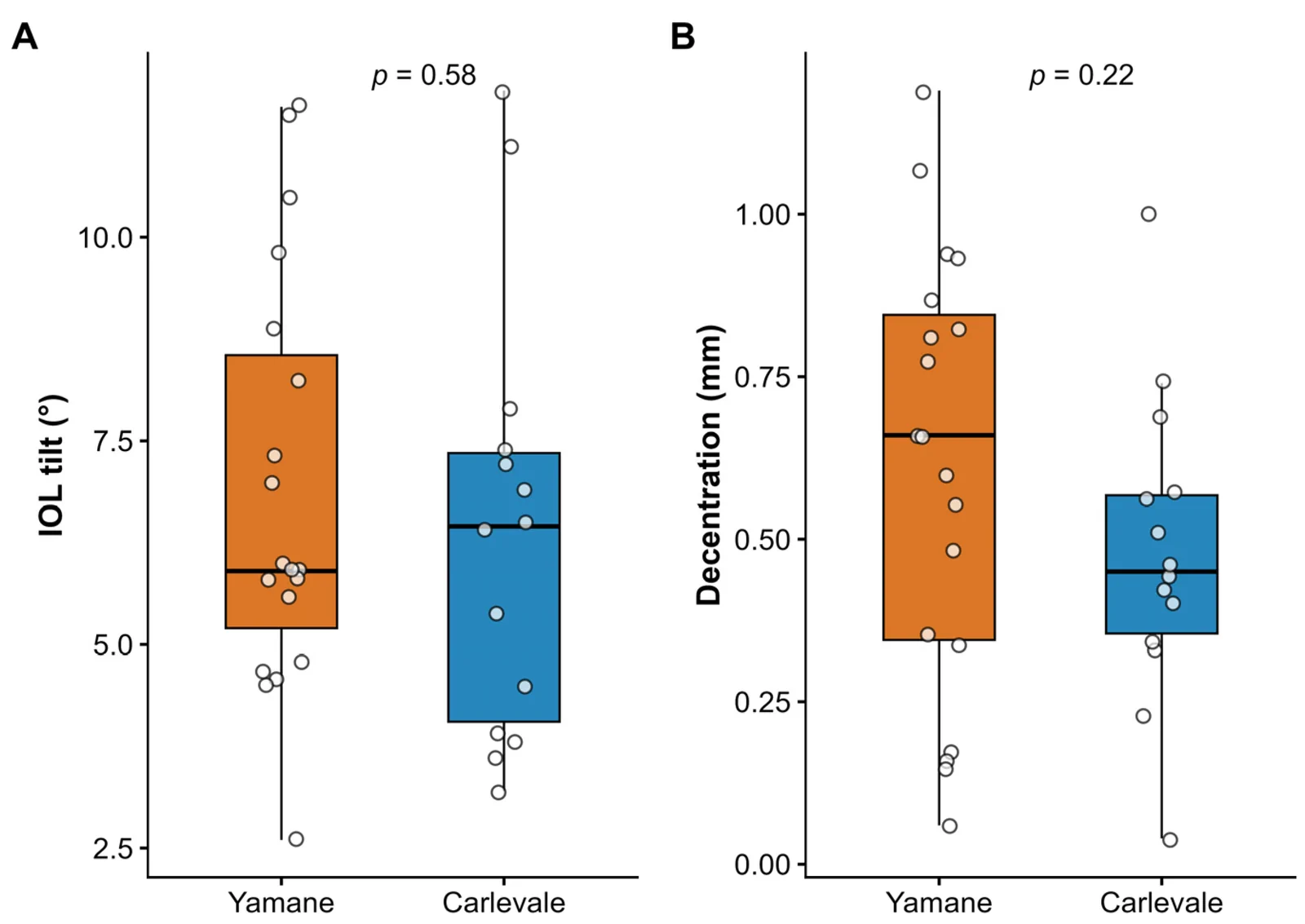
Comparison of intraocular lens (IOL) tilt and decentration between Yamane and Carlevale techniques measured with second-generation swept-source anterior segment OCT. (**A**) Box plot of IOL tilt (degrees), showing no statistically significant difference between groups (*p* = 0.58). (**B**) Box plot of IOL decentration (millimetres), demonstrating no statistically significant difference between groups (*p* = 0.22).

AS-OCT measurements were performed only in patients who completed the 6-month follow-up and for whom high-quality scans were available. In a subgroup of 33 eyes of consecutive patients who attended the 6-month visit (19 Yamane and 14 Carlevale), objective IOL positioning was analyzed using second-generation swept-source AS-OCT (Casia 2, Tomey, Nagoya, Japan) at six months postoperatively. The mean tilt was 6.9° in the Yamane group and 6.4° in the Carlevale group (*p* = 0.58; Figure 3). Regarding decentration, mean values were 0.61 mm for the Yamane technique and 0.48 mm for the Carlevale IOL (*p* = 0.22).

### 3.4. Visual Outcomes

Both groups achieved a significant improvement in mean best-corrected visual acuity (BCVA) from baseline (*p* < 0.001). At baseline, the mean BCVA was 0.793 ± 0.679 logMAR in the Yamane group and 0.504 ± 0.399 logMAR in the Carlevale group (*p* = 0.066). Following the intervention, functional success was high in both groups, with the Carlevale cohort showing an average gain of 0.28 ± 0.39 logMAR.

**Figure 4:**
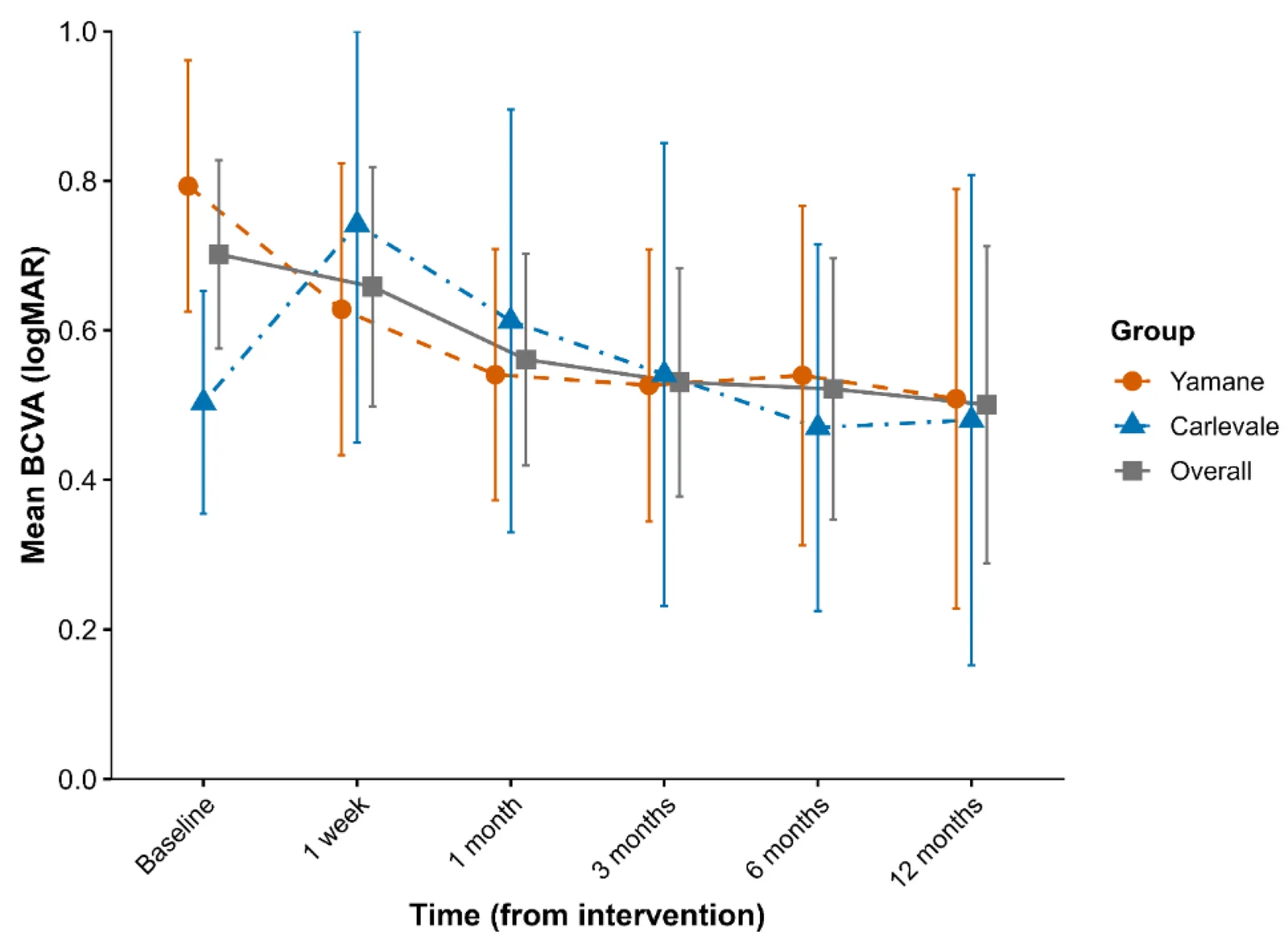
Longitudinal visual acuity outcomes over time. Line plot showing mean best-corrected visual acuity (BCVA, logMAR) at baseline, 1 week, 1 month, 3 months, 6 months, and 12 months postoperatively for the Yamane group, Carlevale group, and total cohort (Overall). Values refer to the available data at each timepoint; the number of eyes varies across timepoints. BCVA, best-corrected visual acuity; logMAR, logarithm of the minimum angle of resolution.

Although this difference did not reach the standard threshold for statistical significance (*p* = 0.066), the Yamane cohort presented with a higher mean logMAR value, indicating a trend toward poorer baseline vision compared to the Carlevale cohort. Both surgical techniques yielded significant improvements in visual function (Figure 4).

In the unadjusted analysis, the Carlevale technique was associated with a smaller improvement in BCVA at 1 week compared with Yamane (ΔlogMAR +0.42, 95% CI +0.06 to +0.79; *p* = 0.023). After adjustment for age, axial length and baseline BCVA, the estimate was attenuated and no longer statistically significant (+0.24, 95% CI −0.14 to +0.63; *p* = 0.213). A similar pattern was observed at 1 month (unadjusted +0.53, 95% CI +0.22 to +0.83, *p* = 0.001; adjusted +0.22, 95% CI −0.10 to +0.53, *p* = 0.171). Worse baseline BCVA was strongly associated with greater improvement at both time points (−0.05 per 0.1 baseline logMAR increase, 95% CI −0.08 to −0.03, *p* < 0.001 at 1 week; −0.06, 95% CI −0.08 to −0.04, *p* < 0.001 at 1 month), whereas age and axial length were not significantly associated with visual outcome.

### 3.5. Postoperative Complications

No intraoperative complications occurred in either group. On the first postoperative day, 59.4% of the Yamane group and 74.2% of the Carlevale group were free of complications (*p* = 0.286). Corneal edema was observed in 16% of eyes in the Yamane group and 13% of eyes in the Carlevale group; these cases reached nearly complete resolution in both cohorts within one week.

**Figure 5:**
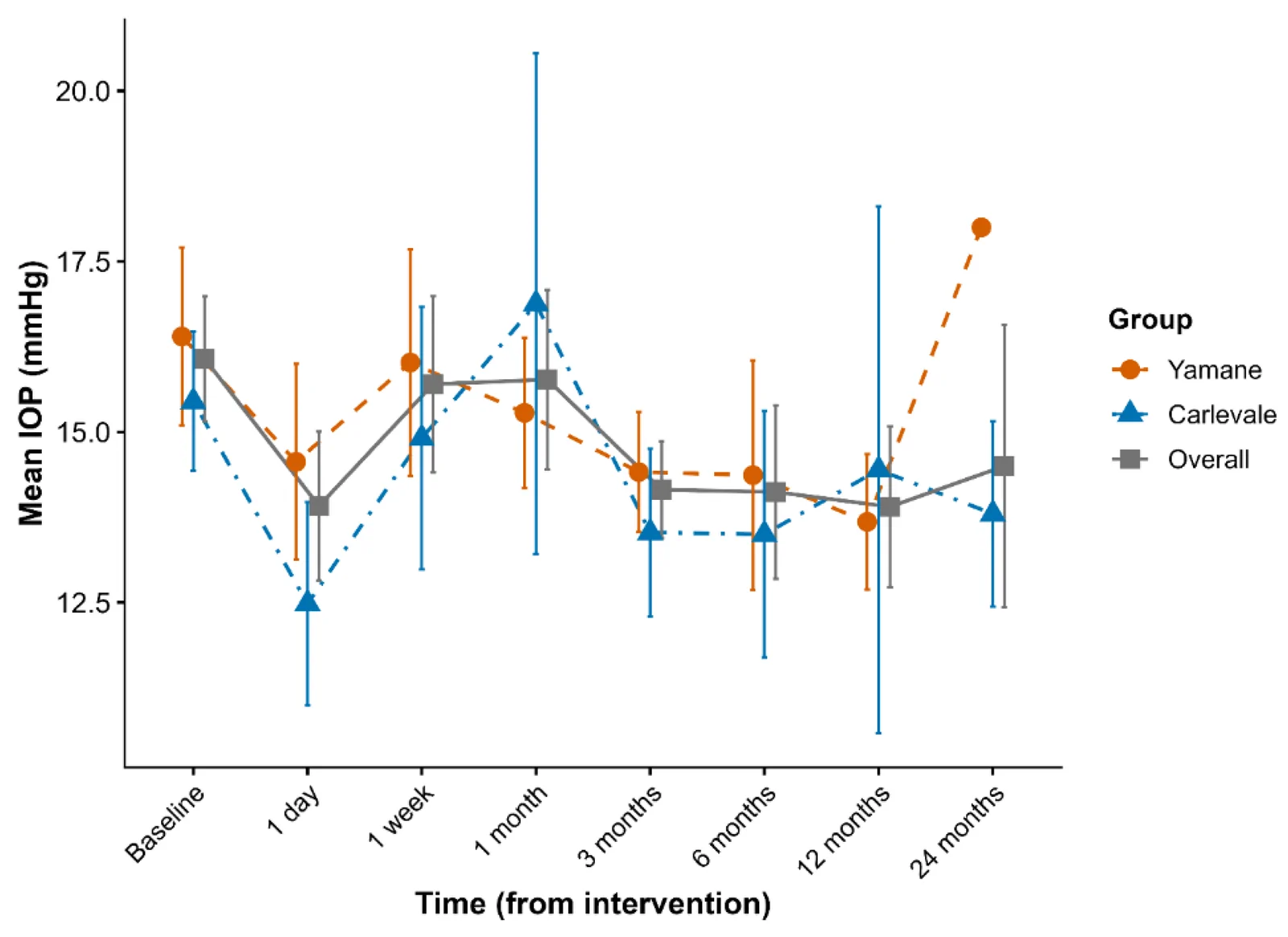
Longitudinal intraocular pressure (IOP) outcomes over time. Line plot showing mean IOP (mmHg) at baseline, 1 day, 1 week, 1 month, 3 months, 6 months, 12 months, and 24 months postoperatively for the Yamane group, Carlevale group, and total cohort (Overall). Values refer to the available data at each timepoint; the number of eyes varies across timepoints.

Regarding intraocular pressure (IOP), mean IOP remained within the physiological range throughout follow-up in both groups, with no clinically relevant differences between the two techniques (Figure 5). Ocular hypertension (IOP > 25 mmHg) was recorded in 7.2% of the Yamane group, whereas no cases were observed in the Carlevale group. Conversely, the Carlevale cohort presented one case of ocular hypotony (IOP < 6 mmHg) associated with choroidal detachment.

Another common early finding was an anterior chamber blood Tyndall effect, noted in nine cases (13%) in the Yamane group and two cases (6%) in the Carlevale group, all of which resolved spontaneously after one week without surgical revision.

**Figure 6:**
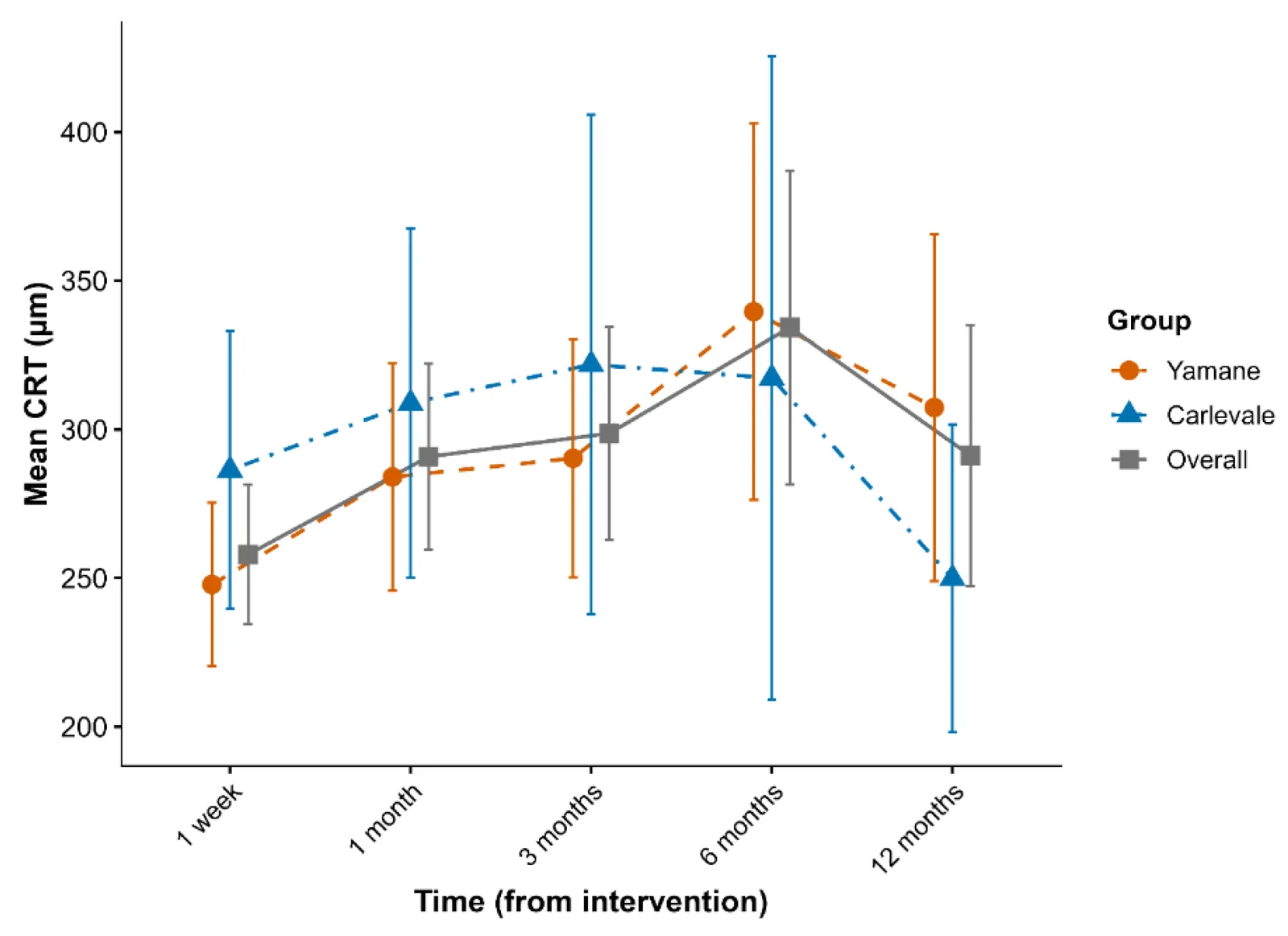
Longitudinal central retinal thickness (CRT) outcomes over time. Line plot showing mean CRT (µm) at 1 week, 1 month, 3 months, 6 months, and 12 months postoperatively for the Yamane group, Carlevale group, and total cohort (Overall). Values refer to the available data at each timepoint; the number of eyes varies across timepoints.

The incidence of cystoid macular edema (CME), as detected by OCT, tended to increase over time in both groups. At one month, CME affected 13.1% of Yamane eyes and 11.5% of Carlevale eyes. By the six-month visit, the prevalence rose to 23.1% in the Yamane group and 15.4% in the Carlevale group (*p* = 0.604). Mean central retinal thickness (CRT) followed a parallel trend in both groups, increasing progressively up to six months and then decreasing at twelve months (Figure 6).

Other long-term complications included retinal detachment, recorded within 12 months in 3 eyes (4.4%) of the Yamane group and 2 eyes (6.5%) in the Carlevale group. Crucially, no instances of the IOL being “flipped upside down” or requiring surgical repositioning due to secondary luxation were reported during the study period.

## 4. Discussion

The management of intraocular lens (IOL) fixation in the absence of capsular support remains a complex surgical challenge with no universally accepted “gold standard” [13]. This study provides a comparative analysis of the Yamane flanged technique and the Carlevale IOL, two sutureless approaches designed to overcome the long-term risks of traditional sutured fixation, such as suture erosion or breakage and late dislocation.

### 4.1. Visual Rehabilitation and Baseline Disparity

In this cohort, both techniques allowed for significant visual rehabilitation. However, a notable finding was the disparity in preoperative visual status: the Yamane group presented with a higher mean logMAR value compared to the Carlevale group. While the Yamane group appeared to exhibit a numerically higher visual gain compared to the Carlevale group, this finding must be interpreted with caution. The Yamane cohort presented a poorer baseline visual acuity, thereby possessing a larger “room for improvement”. Despite the difference in the slope of recovery, the final postoperative BCVA was comparable between groups, confirming that both methods are effective for visual rehabilitation in complex eyes. As noted by Guerin et al. [14], while different secondary fixation techniques can achieve comparable final visual outcomes, the starting clinical condition and associated comorbidities heavily influence the recovery curve.

### 4.2. Anatomical Stability and the Precision of AS-OCT Measurement

A key strength of this study was the objective quantification of IOL stability using second-generation swept-source AS-OCT (Casia 2, Tomey, Nagoya, Japan). As our department is a tertiary referral center, many patients live far from the hospital and are followed by their local ophthalmologist after the early postoperative period; it was therefore not always possible to perform SS AS-OCT at 6 months. AS-OCT was performed in the consecutive patients who attended the 6-month visit at our center (n = 33).

Both the Yamane and Carlevale techniques achieved high anatomical stability, with mean tilt values of 6.9° and 6.4°, respectively, and mean decentration values of 0.61 mm and 0.48 mm. While our tilt values are slightly higher than the 3.8° median reported in some meta-analyses [15] for flanged techniques, they align more closely with recent studies using similar technology, such as Schranz et al. [16], who reported tilt values of 7.67° for the Yamane technique and 6.45° for the Carlevale IOL.

This discrepancy with older literature can be primarily attributed to the advanced measurement methodology provided by the Casia 2 from Tomey. Most previous clinical studies relied on manual measurements or secondary image-analysis software, which are inherently operator-dependent and may underestimate the true degree of IOL displacement. In contrast, the Casia2 utilizes inbuilt automatic software that models the IOL position over 360 degrees, providing operator-independent measurements of tilt and decentration. Furthermore, in cases where the automatic detection is not perfectly aligned, the system allows for a semi-automatic analysis. By manually identifying three points on the anterior surface and three points on the posterior surface of the IOL, the software automatically calculates the tilt and decentration. This dual-modality approach eliminates external influences from the operator or secondary analysis software, thereby providing high-resolution, objective, and highly reproducible measurements.

Therefore, the higher tilt values observed in this cohort likely reflect a more precise and standardized assessment of the IOL’s true position in the posterior chamber rather than a lower surgical accuracy.

Notably, the data seem to confirm a trend toward better centration with the Carlevale IOL (0.48 mm vs. 0.61 mm for Yamane), but given the small sample, these findings do not allow conclusions about great differences between the techniques.

The numerical differences in tilt and decentration between the two groups were small and not statistically significant. Given the limited sample, this study can neither demonstrate nor exclude differences between the two techniques, and it was not designed to test equivalence. Design-related features of the Carlevale IOL, such as its T-shaped haptic plugs that provide a “self-blocking” mechanism within the scleral bed [17], are plausible determinants of IOL stability, but this hypothesis needs to be tested in larger prospective studies.

### 4.3. The Inflammatory Profile and CME Progression

One of the most clinically relevant findings was the progressive increase in cystoid macular edema (CME) during follow-up. At six months, the incidence reached 23.1% in the Yamane group and 15.4% in the Carlevale group. While the difference was not statistically significant (*p* = 0.604), the higher rate in the Yamane group aligns with findings by Ishikawa et al. [18], who reported that flanged techniques carry an increased risk of CME (13% at 3 months) compared to conventional sutured methods.

Moreover, our results align more closely with recent high-sensitivity data, such as Bontemps et al. [13], who reported rates as high as 31.2% for the FIL-SSF lens. The elevated incidence observed in this cohort may be largely attributed to our stringent OCT-based diagnostic criteria. In this study, we classified any presence of small intraretinal fluid (IRF) cells as CME, including very early or subclinical edematous changes. Many earlier clinical studies may have only recorded “clinically significant” CME—edema that resulted in measurable vision loss or macro-cystic changes—thereby excluding these minor fluid accumulations. By capturing these early findings, which may lack immediate clinical significance but reflect a low-grade inflammatory state, recent data provide a more sensitive assessment of the uveal response to secondary IOL fixation than previously reported in the literature.

Chronic low-grade inflammation in the Yamane group might be triggered by subclinical pseudophacodonesis or iris chafing from the IOL flanges [18]. The relatively lower CME rate in our Carlevale cohort (15.4%), despite their significantly longer axial length (26.9 mm vs. 23.8 mm for Yamane; *p* < 0.001), supports the theory that its posteriorly angled haptics and stable scleral anchoring may reduce iris-haptic interaction and subsequent inflammation [17]. These mechanisms are hypotheses: postoperative inflammation was not measured quantitatively, and the study cannot establish that the observed numerical differences were caused by IOL design.

### 4.4. Early and Late Postoperative Complications

Regarding early and late complications, the safety profiles of both techniques were characterized by generally mild and self-limiting events in the immediate postoperative period.

A noteworthy finding in our series was the difference in the incidence of an anterior chamber blood Tyndall effect on the first postoperative day, which was observed in 13% of the Yamane group compared to only 6% of the Carlevale group. Although these cases reached spontaneous resolution within one week without the need for surgical revision, this disparity reflects the distinct mechanical nature of the two procedures. The higher incidence of anterior chamber blood Tyndall effect in the Yamane cohort is likely attributable to the intensive intraocular maneuvers and mechanical friction between the needles and the posterior surface of the iris during haptic externalization. The double-needle technique requires high surgical accuracy for symmetric cannula insertion and haptic docking, often resulting in repeated iris-needle contact that triggers a transient breakdown of the blood-aqueous barrier [18]. In contrast, the Carlevale IOL utilizes posteriorly angled haptics and a unique T-plug design that may be less traumatic to the iris-ciliary body complex during externalization, despite the requirement for a slightly larger initial incision [17,19]. In our opinion this lower early inflammatory response in the Carlevale group is consistent with its intended design to minimize iris chafing and uveal irritation [17].

The early safety profile was further differentiated by intraocular pressure (IOP) fluctuations. We recorded ocular hypertension (IOP > 25 mmHg) in 7.2% of Yamane eyes on day one, whereas no such cases were found in the Carlevale group. This transient elevation may be linked to the aforementioned pigment dispersion or iris trauma during the flanged fixation process. Conversely, the Carlevale cohort presented one case of ocular hypotony associated with choroidal detachment. As documented in the literature, procedures involving larger sclerotomies or the creation of scleral flaps, such as the Carlevale technique, carry a higher inherent risk of transient wound leakage or hypotony if the seal is not initially watertight [13,20].

The incidence of early corneal edema was comparable between the two groups (16% Yamane vs. 13% Carlevale) and resolved rapidly within the first postoperative week. This indicates that both sutureless techniques are relatively sparing of the corneal endothelium, provided the IOL is maintained in a physiological posterior chamber position, thereby avoiding the high rates of endothelial decompensation historically associated with anterior chamber IOLs [13,17].

Regarding long-term outcomes, the risk of retinal detachment remained low but present in both cohorts (4.4% in the Yamane group at 12 months and 2 eyes in the Carlevale group). This reinforces the necessity of performing a meticulous total vitrectomy during secondary IOL fixation, regardless of the specific technique used, to eliminate vitreous traction [13,14,19].

Finally, while no instances of IOL luxation or “flipping” were observed in this study, the long-term risk of haptic extrusion remains a concern for scleral-fixated lenses. Long-term imaging studies, such as those by Schranz et al., have shown significant thinning of the scleral pockets overlying haptic plugs over time [16,20]. This structural change suggests that patients undergoing these procedures require vigilant long-term monitoring to detect late-onset anchor exposure, which could otherwise lead to serious complications such as endophthalmitis [13,19].

### 4.5. Macular Morphological Changes and Intravitreal Injections (IVT)

Longitudinal analysis of Central Retinal Thickness (CRT) revealed a shared evolutionary profile between the two cohorts, characterized by a progressive increase from the early postoperative stages until reaching a peak at the six-month interval. At this timepoint, the Yamane group exhibited a higher mean CRT compared to the Carlevale group. This morphological trend was reflected in a significantly higher therapeutic burden for the Yamane cohort: among patients who developed CME 54.5% in the Yamane group required treatment with IVTs, compared to 33.3% in the Carlevale group. By the 12-month follow-up, a marked reduction in CRT was observed in both groups, suggesting a favorable response to therapeutic interventions and the stabilization of the blood–retinal barrier.

### 4.6. Axial Length Discrepancy and Confounding by Indication

A notable finding in this study was the significant disparity in mean axial length (AL) between the two groups: 23.8 ± 1.63 mm for the Yamane group and 26.9 ± 3.83 mm for the Carlevale group (*p* < 0.001). While some literature suggests a clinical preference for the Carlevale IOL in myopic eyes due to the stability of its T-shaped plugs in a wider ciliary sulcus, showing that the Carlevale lens maintains excellent centration even in eyes with increased ocular dimensions, the discrepancy observed in this cohort likely reflects a confounding by indication inherent to the retrospective study design and to the different clinical scenarios in which each technique was preferentially adopted.

In our department, the Kowa 3-piece lenses (PU6AS) required for the Yamane technique are maintained in consignment stock, though this inventory is limited to standard or “main” dioptric powers. Conversely, Carlevale IOLs are typically ordered on a case-by-case basis for planned procedures. This logistical arrangement directly influenced the surgical decision-making process, particularly in the management of unexpected intraoperative complications.

Our data show that the necessity for IOL implantation following a complicated cataract surgery was significantly higher in the Yamane group (52.2%) compared to the Carlevale group (12.9%; *p* < 0.001). In these emergency scenarios, if the appropriate dioptric power was immediately available in the Kowa consignment stock, the surgeon opted for the Yamane technique to provide immediate visual rehabilitation. Because eyes with extreme axial lengths (such as high myopes) often require non-standard IOL powers that are not kept on-site, these cases were more frequently managed through elective, planned Carlevale implantations. Given that our expert surgeon (L.V.) possesses high and equal proficiency in both techniques, the choice of fixation was driven by the immediate availability of the lens power rather than clinical preference. The surgeon had been performing both techniques before the study period and had comparable experience with each; the choice between them was therefore determined by lens availability and by the urgency of the clinical scenario, not by differing levels of familiarity with the two procedures. This explains the concentration of “standard” axial lengths in the Yamane group and the higher prevalence of longer, more complex eyes in the Carlevale cohort.

Sutureless scleral fixation is recognized as one of the most technically demanding procedures in secondary IOL implantation, carrying a steep learning curve that can significantly influence complication rates. To minimize the impact of this effect on our results, all procedures were performed by a single highly experienced vitreoretinal surgeon (L.V.), and the first 30 cases performed with each technique were excluded from the analysis.

While the Carlevale technique aims to standardize the procedure through a dedicated prosthetic design, the Yamane technique remains highly operator-dependent, particularly regarding the symmetry of sclerotomies and the manual creation of haptic flanges. Our findings suggest that high surgical proficiency is essential for both techniques to ensure precise IOL centration and to mitigate intraoperative risks such as iris trauma or haptic deformation, which are more frequent during the early stages of the learning process.

The Yamane group was also significantly older (median 79 vs. 65 years). This difference most likely reflects the different case mix: Yamane fixation was more often performed as an immediate solution after complicated cataract surgery (52.2% vs. 12.9%), a scenario typical of an elderly cataract population, whereas Carlevale IOLs were more often implanted in a planned fashion for late IOL subluxation or luxation. Because age and axial length were unevenly distributed, both were included as covariates in the multivariable analysis of BCVA. Importantly, because of the differences in age, axial length and surgical indications between the groups, this study compares not only two fixation techniques but also the different clinical scenarios in which each was preferentially adopted. All between-group differences observed in this study, including those in postoperative CME and IOL position, should therefore be interpreted as exploratory associations rather than technique-specific effects.

The findings of this study have practical clinical implications. First, both techniques provided significant visual improvement with satisfactory anatomical stability, supporting their use as reliable options for secondary sutureless fixation when performed by an experienced surgeon. Second, the frequent occurrence of OCT-detected CME may warrant systematic macular OCT monitoring during the first postoperative months after scleral fixation, so that treatment can be started early. Third, automated 360° AS-OCT analysis allows an objective, operator-independent assessment of IOL tilt and decentration in routine practice, and could support future comparisons between IOL designs.

### 4.7. Study Limitations

This study has several limitations that should be acknowledged. First, its retrospective single-center design may have introduced selection bias and limits the generalizability of the findings. Second, no a priori sample size calculation was performed; the groups were unbalanced in size, and the AS-OCT analysis was based on a small subgroup. As our department is a tertiary referral center and many patients are followed by their local ophthalmologist, attendance at the six-month AS-OCT depended mainly on geographic factors; therefore selection bias cannot be excluded, and the AS-OCT findings should be considered exploratory. Third, the groups differed in age, axial length and surgical indications; multivariable adjustment was applied only to BCVA, whereas complications, including CME, and IOL position were compared without adjustment because of the limited number of events and eyes, so residual confounding by indication cannot be excluded and all between-group differences should be considered exploratory. Fourth, CME was defined with sensitive OCT criteria including subclinical forms, which limits comparability with other series, and its grading was retrospective and not masked to the surgical technique, which may have introduced observer bias. Fifth, excluding the first 30 cases of each technique reduces the influence of the learning curve but may introduce temporal selection bias. Sixth, postoperative refractive outcomes were not analyzed in this study and will be the subject of a dedicated analysis.

Finally, although all procedures were performed by a single experienced vitreoretinal surgeon, which reduces inter-surgeon variability, the findings may not be generalizable to surgeons with different levels of experience. Larger prospective multicenter studies are warranted to validate these results.

## 5. Conclusions

Both the Yamane technique and the Carlevale IOL were associated with significant visual improvement and acceptable safety profiles for secondary sutureless scleral fixation in eyes without adequate capsular support. Although in our sample no statistically significant differences were observed in IOL tilt and decentration, the Carlevale IOL showed a trend toward better centration, whereas second-generation swept-source AS-OCT proved to be a valuable objective imaging modality for the postoperative evaluation of scleral-fixated IOLs. Larger prospective multicenter studies are needed to confirm these findings.

The use of second-generation swept-source AS-OCT (Casia 2) allowed an objective, high-resolution, and reproducible assessment of IOL positioning, highlighting its value as a valuable imaging tool for postoperative evaluation of scleral-fixated intraocular lenses.

Given the retrospective design and the heterogeneity of the study population, these findings should be interpreted with caution. Nevertheless, both techniques represent reliable surgical options when performed by experienced surgeons. Larger prospective multicenter studies are needed to further clarify their long-term anatomical and functional outcomes and to establish whether these results are confirmed in comparable populations.

## Data Availability

The original contributions presented in this study are included in the article. Further inquiries can be directed to the corresponding author.
